# Modification of ZnO nanoparticles with silanes enables their application as anticancer agents

**DOI:** 10.1080/07853890.2021.1896916

**Published:** 2021-09-28

**Authors:** Fernanda Marques, Aurel Tǎbǎcaru, Mariana Bușilǎ, Teresa Pinheiro, António P. A. Matos

**Affiliations:** a Centro de Ciências e Tecnologias Nucleares, Instituto Superior Técnico, Universidade de Lisboa, Bobadela, Portugal; b Department of Chemistry, Physics and Environment "Dunarea de Jos", University of Galati, Galati, Romania; c Department of Materials Science and Engineering “Dunarea de Jos”, University of Galati, Galati, Romania; d Institute for Bioengineering and Biosciences, Departamento de Engenharia e Ciências Nucleares, Instituto Superior Técnico, Universidade de Lisboa, Bobadela, Portugal; e Centro de Investigação Interdisciplinar Egas Moniz (CiiEM), Egas Moniz Cooperativa de Ensino Superior, Caparica, Portugal

## Abstract

**Introduction:**

Nanoparticles (NPs) are a wide class of materials that include particulate substances sized less than 100 nm. Inorganic ZnO NPs have found applications in several industrial fields such as the optical, electronic, pharmaceutical and cosmetics [1]. However, in many specific fields the applications are limited, since the particles tend to aggregate/agglomerate due to the hydrophilic nature of the surface. For potential clinical applications, surface modification of ZnO plays a crucial role in the biocompatibility of ZnO NPs [2]. Using organosilane modifying agents, improved hydrophobicity of the resulting ZnO NPs, induced by the non-polar terminal groups, can be achieved, and thus, very small and highly dispersed particles can be obtained. ZnO silanes were found to have antibacterial activity against several pathogens [3]. The NP size highly influenced the antibacterial activity, which increase with decreasing size. The ZnO NPs may induce bacterial cell membrane damage, resulting in bacterial cell death. Motivated by these findings and others reporting anticancer activity for pristine ZnO NPs [4], the aim of this study was to investigate the anticancer activity and mechanism of cell death of silane-modified ZnO nanoparticles against A2780 ovarian cancer cells and evaluate if for the cancer cells a correlation between size and activity was also observed.

**Materials and Mmethods:**

The silane modified ZnO NPs were prepared by the addition of (3-glycidyloxypropyl) trimethoxysilane (GPTMS) as a surface modifier at 0% (G0) and 10% (G3) molar ratio of Si/Zn. The obtained NPs were characterised by high resolution transmission electron microscopy (HRTEM), X-ray diffraction and UV-Vis spectrometry. The cytotoxic activity in ovarian cancer cells were assessed by the MTT colorimetric assay. The morphological cellular alterations were visualised by electron microscopy (TEM).

**Results:**

The silane modified ZnO NPs exhibited significant cytotoxic activity against A2780 ovarian cancer cells. The NP size, *ca.* 13 nm for G0 and *ca*. 3 nm for G3, highly influenced the cytotoxic activity, which increased with decreasing particle size, IC_50_: ∼100 µg/mL(G0) and ∼30 µg/mL(G3). The ZnO silanes affected the cellular integrity by the induction of organelle damage evidenced by TEM.

**Discussion and conclusions:**

Although preliminary, results indicate that ZnO silanes are interesting platforms to explore as anticancer agents. Studies on the ultrastructural level (TEM/SEM) are needed to understand their cytotoxic mechanism and to give clues on their potential targets.
